# Neurodevelopmental Outcomes of Very Low Birth Weight Infants Following Extrauterine Placental Perfusion: A Follow‐Up Study

**DOI:** 10.1111/apa.70101

**Published:** 2025-04-18

**Authors:** Benjamin Kuehne, Martin Hellmich, Eva Heine, Angela Kribs, Katrin Mehler, André Oberthuer

**Affiliations:** ^1^ Division of Neonatology, Faculty of Medicine and University Hospital Cologne, Department of Pediatrics University of Cologne Cologne Germany; ^2^ Faculty of Medicine and University Hospital Cologne, Institute of Medical Statistics and Computational Biology, University of Cologne Cologne Germany; ^3^ Department of Medical Statistics University Medical Center Göttingen Göttingen Germany

**Keywords:** extrauterine placental perfusion, neurodevelopmental outcome, physiological based cord clamping, very low birth weight infants

## Abstract

**Aim:**

Extrauterine placental perfusion (EPP) may be a feasible cord clamping strategy in very low birth weight (VLBW) infants to support neonatal transition. However, the impact of EPP on neurodevelopment remains unclear. The study aimed to compare the effects of EPP with time‐based delayed cord clamping (DCC) on neurodevelopmental outcomes.

**Methods:**

This follow‐up study of the randomised controlled EXPLAIN (Extrauterine Placental Transfusion in Resuscitation of Very Low Birth Weight Infants) trial (ClinicalTrials.gov Identifier: NCT03916159) was conducted at a tertiary perinatal centre from 2021 to 2023. Antenatally randomised VLBW infants received either EPP or DCC (> 30 s). Neurodevelopment was assessed at 24 months of corrected age using the Bayley Scales of Infant and Toddler Development, Third Edition. Data analysis was intention‐to‐treat.

**Results:**

Of 59 infants enrolled, 54 (92%) participated in the follow‐up (27 EPP, 27 DCC). Median age at assessment was 24.3 months (range 23.5–25.0); 28 (52%) were male. Infant characteristics and short‐term outcomes were similar between groups. No relevant differences were observed in median cognitive, motor or language scores or in rates of cerebral palsy, hearing, or visual impairment.

**Conclusion:**

The neurodevelopment of the VLBW infants who received EPP and DCC was comparable, suggesting that EPP may be a viable alternative.

**Abbreviations**
BSID III: Bayley Scales of Infant and Toddler Development Third Edition
CPAP: continuous positive airway pressure
DCC: delayed cord clamping
EPP: extrauterine placental perfusion
IVH: intraventricular haemorrhage
PBCC: physiological based cord clamping
SGA: small for gestational age
VLBW: very low birth weight

**Summary**

- This study was needed to evaluate whether extrauterine placental perfusion could be a feasible alternative to delayed cord clamping in very low birth weight infants requiring respiratory support at birth.
- Neurodevelopmental outcomes at 24 months of corrected age were found to be comparable between the two groups.
- These findings suggest that extrauterine placental perfusion may be considered as an alternative cord clamping strategy in clinical practice.

## 1 Introduction

Delayed umbilical cord clamping (DCC) has become a standard practice for supporting the neonatal transition of preterm infants as it has been shown to improve survival [1, 2]. However, data on long‐term neurodevelopmental outcomes in preterm infants receiving different cord clamping strategies are still limited [3]. Current recommendations state that infants who are not breathing sufficiently after birth should not receive DCC [4]. Most previous studies of DCC have therefore excluded infants who required treatment for respiratory insufficiency immediately after birth [1], which is a common condition in many very low birth weight (VLBW) infants [5]. Animal studies have suggested that ensuring adequate lung aeration prior to umbilical cord clamping may be critical for the benefits associated with DCC [6, 7]. Consequently, the decision of when to clamp the cord should not be based on time alone. Instead, it should depend on the infant's respiratory effort and the achievement of lung aeration. This approach is known as physiological‐based cord clamping (PBCC). The majority of VLBW infants require respiratory support with continuous positive airway pressure (CPAP) during the transition to achieve adequate lung aeration. Providing effective respiratory support while maintaining an intact umbilical cord can be challenging. The process requires careful coordination to ensure adequate aeration, maintain the infant's body temperature, and work within the limited space available. In Caesarean births, maintaining sterility in the theatre adds complexity [8]. There is ongoing debate about whether cord milking may increase the risk of intraventricular haemorrhage (IVH) in VLBW infants [9]. Several studies have focused on the effects of PBCC on the incidence of severe IVH and other long‐term affecting neonatal complications [10, 11]. Since PBCC has been shown to improve stabilisation and oxygenation during the neonatal transition in preterm infants, it may also positively affect neurodevelopmental outcomes [12, 13]. The aim of this follow‐up study was to assess the effects of EPP versus DCC at birth on the neurodevelopmental outcomes of the infants at 24 months of corrected age.

## 2 Methods

This study presents 2‐year follow‐up data from infants enrolled in the single‐center, randomised controlled trial, called Extrauterine Placental Transfusion in Resuscitation of Very Low Birth Weight Infants (EXPLAIN). This study was conducted at the University Hospital of Cologne, Germany, between 2019 and 2021. Infants born via Caesarean delivery with birth weight < 1500 g and gestational age > 23 + 6 weeks of gestation were eligible for the EXPLAIN trial. The exclusion criteria for the primary study included vaginal delivery, foetal or maternal risk, placental abruption or placenta previa with haemorrhage, placental anomalies, monochorionic multiples, and congenital anomalies.

In total, 59 pregnant women with 49 singleton and 10 twin VLBW infants were randomised. Infants were allocated to receive either extrauterine placental perfusion (EPP) approach or time‐based DCC of at least 30 s following Caesarean delivery. Infants in the EPP group were transferred to the resuscitation table with an intact umbilical cord and received CPAP respiratory support while maintaining prolonged placental perfusion. During the neonatal transition, these infants showed improved peripheral and cerebral oxygenation. However, there was no difference in the primary outcome of mean haematocrit during the first 24 h after birth [12]. Other neonatal and maternal outcomes also did not differ significantly between groups.

Eligible infants from the EXPLAIN trial were enrolled in a structured follow‐up program for VLBW infants in accordance with national guidelines. Follow‐up visits were scheduled at 3, 6, 12, and 24 months of corrected age and included a physical examination with detailed neurological assessment, as well as interval history. In addition, all study participants were seen by physiotherapists, ophthalmologists and otolaryngologists at regular intervals during the first year after discharge. If necessary, treatment measures were taken.

All study data were collected from maternal and infant medical records and managed using REDCap electronic data capture tools hosted at University of Cologne through the corrected age of 24 months [14, 15]. REDCap (Research Electronic Data Capture) is a secure, web‐based software platform designed to support data capture for research studies.

Neurodevelopmental outcomes were assessed at 24 months of corrected age using the Bayley Scales of Infant and Toddler Development, Third Edition (BSID III), in the validated German translation [16]. The BSID III is routinely used in the neonatal follow‐up programme at our centre as part of the national quality assurance protocol for VLBW infants care and was therefore defined as the prespecified secondary outcome of this follow‐up study. For infants who did not undergo BSID III testing, the presence of cerebral palsy, auditory impairment, or visual impairment was recorded instead. Blinded investigators conducted BSID III testing between 2021 and 2023, and results were shared with the study authors by the respective clinical sites, as not all assessments were performed at the University Hospital of Cologne.

The BSID III provides age‐standardised composite scores for cognitive, language, and motor development, with a mean score of 100. The language scale consists of receptive and expressive communication subtests, while the motor scale includes fine and gross motor subtests. Mild developmental delay was defined as a score between 70 and 84, moderate delay as 55–69, and severe delay as scores below 55.

Baseline patient's characteristics were recorded, including anthropometric data for weight, length, and head circumference, presented as age‐adjusted percentiles using national reference charts [17, 18].

Small for gestational age (SGA) was defined as a birth weight below the 10th percentile. IVH was staged according to the criteria of Papile et al. [19].

### 2.1 Statistical Analysis

Statistical analysis was performed using IBM SPSS Statistics for Macintosh, Version 27 (IBM Corp, New York, USA). Distributions of variables are summarised as mean and standard deviation (SD), as median and interquartile range (IQR) or absolute and relative frequencies. Differences between groups were compared by Mann–Whitney *U*‐test (ordinal outcomes) or chi‐square, Fisher's exact test or Barnard's test (nominal outcomes). Twin‐birth‐induced stochastic dependence was deemed negligible. Intention‐to‐treat analysis was used. The sample size calculation was based on the trial primary outcome of mean haematocrit, as has been previously described [12]. As this follow‐up study primarily reports secondary outcomes, no formal prospective sample size calculation was performed for the neurodevelopmental endpoints. A two‐tailed *p*‐value < 0.05 was considered statistically significant. To account for participant mortality, sensitivity analysis was performed by assigning the lowest possible score in all three assessment categories: cognitive, motor and language scales.

### 2.2 Ethics

Ethical approval was obtained from the ethics committee of the University of Cologne (18–232). Parents of infants included in the study received verbal and written information, and written consent was obtained. The study was registered in ClinicalTrials.gov (NCT03916159).

## 3 Results

**FIGURE 1:**
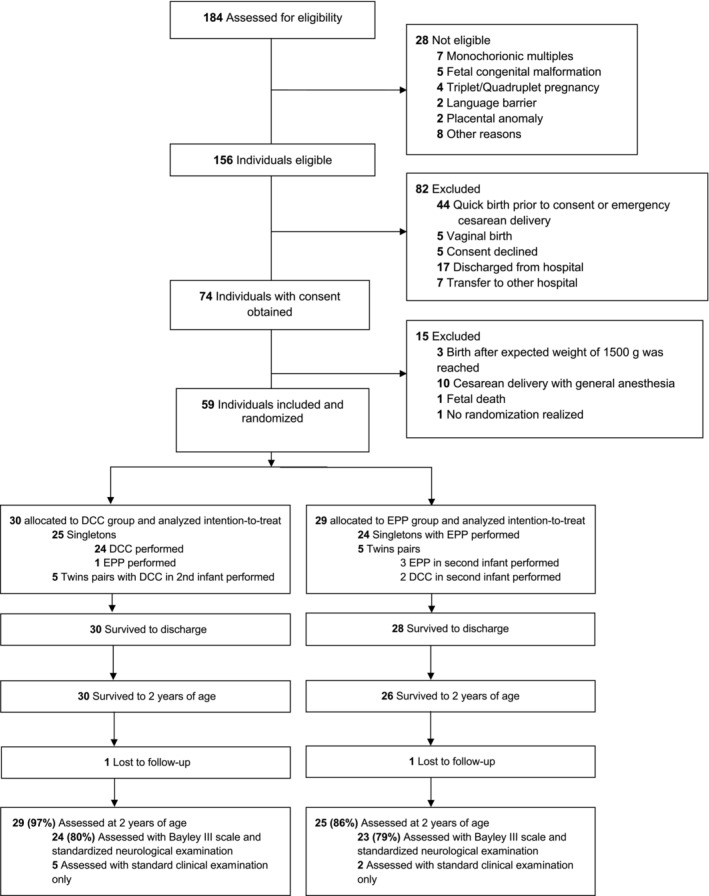
Study flow chart of EXPLAIN follow‐up study.

Of the 59 infants originally enrolled in the EXPLAIN trial, 54 (91.5%) participated in this follow‐up study at 24 months of corrected age. Five infants were not evaluated, because they either died, their families could not be contacted, or their parents refused to participate (Figure 1). Seven of the 54 participants underwent only a standard clinical examination without BSID III testing.

Infant characteristics and short‐term outcomes were similar between the groups, except for a higher incidence of maternal pre‐eclampsia in the EPP group (*p* = 0.04) (Table 1).

**TABLE 1:** Basic patient's characteristics.

	EPP (*n* = 25)	DCC (*n* = 29)	*p*
Gestational age, mean ± SD (days), week+days	28 + 1 ± 14.74	28 + 2 ± 17.12	0.88
Birth weight, mean ± SD, grams	1012.84 ± 270.97	990.55 ± 308.53	0.78
Sex			1.00
Female, *n* (%)	14 (56.0)	16 (55.2)	N/A
Male, *n* (%)	11 (44.0)	13 (44.8)	N/A
Multiple birth, *n* (%)	4 (16.0)	5 (17.2)	1.00
Preeclampsia, *n* (%)	2 (8.0)	9 (31.0)	0.04
HELLP, *n* (%)	0 (0)	3 (10.3)	0.24
SGA, *n* (%)	3 (12.0)	10 (34.5)	0.06
Time to umbilical cord clamp, mean ± SD, sec	494.26 ± 178.29	39.14 ± 8.36	< 0.001
Intraventricular haemorrhage			0.25
Grade 1, *n* (%)	3 (12.0)	6 (20.7)	N/A
Grade 2, *n* (%)	1 (4.0)	0 (0)	N/A
Grade ≥ 3, *n* (%)	2 (8.0)	0 (0)	N/A
Periventricular Leukomalacia, *n* (%)	1 (4.0)	0 (0)	0.46
Maternal age, median [IQR]	32.0 [28.5–37.0]	35.0 [29.5–36.5]	0.47
High school degree (mother), *n* (%)	14/25 (56.0)	16/26 (61.5)	0.78
Single mother, *n* (%)	2/22 (9.1)	4/24 (16.7)	0.67
Multilingualism, *n* (%)	7/21 (33.3)	9/26 (34.6)	1.00
Corrected age at follow‐up, mean ± SD, month	24.7 (2.0)	25.0 (1.4)	0.22

*Note:* Variables are described as mean ± SD, median [IQR] or absolute (relative) frequency.

Abbreviations: HELLP, hemolysis, elevated liver enzymes and low platelet count; IQR, interquartile range; SD, standard deviation.

No relevant nor statistically significant differences were observed between the EPP and DCC groups in cognitive (*p* = 0.60), language (*p* = 0.659) or motor (*p* = 0.117) scores (Table 2). Similarly, no relevant differences were noted in the rates of mild, moderate or severe developmental delay across these domains. Rates of cerebral palsy, visual impairment and auditory impairment were also comparable between groups.

**TABLE 2:** Neurodevelopmental outcomes.

	EPP group	DCC group	*p*
Cognitive composite score, median [IQR]	95 [80–115] (*n* = 23)	90 [85–110] (*n* = 24)	0.60
Cognitive score < 85, *n* (%)	6 (26.1)	5 (20.8)	0.74
Cognitive score < 70, *n* (%)	3 (13.0)	1 (4.2)	0.35
Cognitive score < 55, *n* (%)	0 (0)	0 (0)	N/A
Language composite score, median [IQR]	91 [60–114] (*n* = 19)	91 [81–106] (*n* = 16)	0.66
Language score < 85, *n* (%)	9 (47.4)	6 (37.5)	0.73
Language score < 70, *n* (%)	5 (26.3)	1 (6.3)	0.19
Language score < 55, *n* (%)	3 (15.8)	0 (0)	0.23
Motor composite score, median [IQR]	96 [73–107.5] (*n* = 21)	82 [70.75–102.25] (*n* = 20)	0.12
Motor score < 85, *n* (%)	6 (28.6)	10 (50.0)	0.21
Motor score < 70, *n* (%)	2 (9.5)	3 (15.0)	0.66
Motor score < 55, *n* (%)	1 (4.8)	0 (0)	1.00
Cerebral palsy, *n* (%)	1/25 (4.0)	0/29 (0)	0.46
Auditory impairment, *n*/total (%)	1/24 (4.2)	0/27 (0)	0.47
Visual impairment, *n*/total (%)	3/25 (12.0)	1/29 (3.4)	0.33
Weight percentile at follow‐up, mean ± SD, %	*n* = 19 33.2 ± 25.7	*n* = 24 30.7 ± 32.1	0.27
Length percentile at follow‐up, mean ± SD, %	*n* = 19 38.3 ± 31.5	*n* = 21 30.1 ± 27.9	0.42
Head circumference percentile at follow‐up, mean ± SD, %	*n* = 19 30.7 ± 27.2	*n* = 23 20.9 ± 21.8	0.32

*Note:* Variables are described as mean ± SD, median [IQR] or absolute (relative) frequency.

Abbreviations: IQR, interquartile range; N/A, not applicable; SD, standard deviation.

Sensitivity analysis, including deceased patients (*n* = 3), assigned the lowest possible scores for cognitive, motor and language scales. This analysis confirmed no relevant or statistically significant differences in cognitive (*p* = 0.845), language (*p* = 0.271) or motor (*p* = 0.479) scores between the EPP and DCC groups.

## 4 Discussion

In this follow‐up study of the EXPLAIN trial, we compared the neurodevelopmental outcomes of VLBW infants who received either physiological‐based cord clamping using the EPP approach or time‐based DCC. We found no relevant differences in the cognitive, language or motor scores at 24 months of corrected age. There were also no relevant differences in the rates of cerebral palsy, visual or hearing impairment in infants randomised to receive EPP versus DCC at birth. The results from this follow‐up study, combined with previous findings [12] therefore indicate that EPP is a viable alternative to the currently recommended time‐based DCC procedure. By maintaining the placental circulation during lung aeration, EPP may help stabilise cardiac output and prevent disturbances in cerebral blood flow. Notably, neonatal transition data from the EXPLAIN trial suggest that EPP may improve both peripheral and cerebral oxygenation [12]. Limited evidence suggests that reduced oxygenation during this critical period in preterm infants may be associated with an increased risk of adverse neurodevelopmental outcomes [20, 21]. However, our study results do not reflect this trend.

Prolonged placental perfusion could further optimise tissue perfusion and influence cerebral hemodynamics, potentially reducing the risk of moderate and severe IVH [22], a key predictor of poor neurodevelopmental outcomes [23]. However, since the overall incidence of moderate to severe IVH in VLBW infants at our center is relatively low [24], an association cannot be assessed here due to the small study population of this follow‐up study.

A study in a population of very immature VLBW infants could not confirm the hypothesis that prolonged cord clamping with PBCC results in smoother cardiorespiratory adaptation or improved short‐term outcomes, including IVH rate, compared to cord milking [10]. At all, there is very limited data on the neurocognitive outcomes of different cord clamping strategies in preterm infants. El‐Naggar et al. [25] found comparable cognitive, motor and language scores at 36 months of corrected age between infants who received umbilical cord milking and early cord clamping. Another study showed significantly improved motor function at 18–22 months of corrected age in infants who underwent a DCC compared to early cord clamping [26]. Due to different assessment ages, comparability with our 24‐month results is limited. However, the mean composite scores for cognitive, language, and motor skills in our study cohort remain within the expected range for VLBW infants according to the German norm outside of a study setting [27]. The Cord Pilot Trial, which compared PBCC with immediate cord clamping in preterm infants, suggested a reduced risk of death or adverse neurodevelopmental outcome at 2 years of age. However, there was no clear evidence of reduced moderate or severe impairment on the BSID III in the PBCC group [28]. The as yet unreported secondary data on long‐term neurodevelopmental outcomes from the meta‐analysis by Seidler et al. [29] may provide valuable data on the use and effect of duration in deferring cord clamping in VLBW infants.

## 5 Strengths and Limitations

This is, to our knowledge, the first study reporting 24‐month neurodevelopmental outcomes of EPP versus DCC with a high follow‐up compliance rate of > 85%. Neurodevelopmental assessment was performed by blinded, qualified investigators.

However, several factors may affect Bayley test results: First, there was a high proportion of children who grew up in a multilingual environment, potentially influencing language scores. Also, 17% of EPP and 33% of DCC group children could not be assessed in expressive and receptive language due to insufficient participation in language tasks. These are important factors that may limit the validity of the study results. Second, as this study reports secondary outcomes from the previous EXPLAIN study, the primary limitation lies in the potential underpowering of the study to detect potential differences in neurocognitive outcomes between groups. There are also missing data from deceased individuals and infants who were lost to follow‐up. Additionally, the influence of local practice patterns and expertise in EPP and DCC implementation may have contributed to the observed outcomes, warranting caution in extrapolating these findings to other clinical settings. A potential underestimation of developmental delay by using standardised Bayley‐III scores [30] and differences between local European and American norms for all subtests of the Bayley‐III scores are other important limitations of our study results [31]. Follow‐up at school age would allow more refined testing and detection of subtle intelligence quotient differences.

## 6 Conclusion

In summary, our study showed that EPP resulted in neurocognitive outcomes comparable to time‐based DCC at 24 months of corrected age. EPP represents a promising alternative, particularly when time‐based DCC delays the initiation of respiratory support or when the combination of time‐based DCC with CPAP respiratory support is not feasible. These advantages, combined with the potential for enhanced cerebral oxygenation during neonatal transition, warrant further randomised controlled trials designed and powered to assess long‐term outcomes.

## Author Contributions

**Benjamin Kuehne:** conceptualization, investigation, funding acquisition, writing – original draft, methodology, project administration, data curation, formal analysis, visualization, writing – review and editing. **Martin Hellmich:** conceptualization, supervision, project administration, formal analysis, writing – review and editing, methodology. **Eva Heine:** investigation, writing – review and editing. **Angela Kribs:** conceptualization, writing – review and editing, supervision, investigation, project administration. **Katrin Mehler:** investigation, writing – review and editing. **André Oberthuer:** conceptualization, investigation, writing – review and editing, supervision, project administration.

## Conflicts of Interest

The authors declare no conflicts of interest.
